# Supplementary material for: Cancer Tissue Classification Using Supervised Machine Learning Applied to MALDI Mass Spectrometry Imaging
Source: Cancers (Basel). 2021 Oct 27;13(21):5388. doi: 10.3390/cancers13215388 (PMC8582378; doi:10.3390/cancers13215388)
Supplement: Supplementary file 1 [file cancers-13-05388-s001.zip › cancers-1374076-supplementary.pdf]

Supplementary Material: Cancer Tissue Classification Using Supervised Machine Learning Applied to MALDI Mass Spectrometry Imaging

Paul Mittal, Mark R. Condina, Manuela Klingler-Hoffmann,Gurjeet Kaur, Martin K. Oehler, Oliver M. Sieber, Michelle Palmieri, Stefan Kommoss, Sara Brucker, Mark D. McDonnell and Peter Hoffmann

	1 2	3 4	5 6	7 8	9 10	11 12	13 14	15 16	17 18	19 20	21 22	23 24
**A**	P1 Tumour (X2)	P2 Tumour (X2)	P3 Tumour (X2)	P4 Tumour (X2)	P5 Tumour (X2)	P6 Tumour (X2)	P85 Tumour (X2)	P86 Tumour (X2)	P87 Tumour (X2)	P88 Tumour (X2)	P89 Tumour (X2)	P90 Tumour (X2)
**B**	P7 Tumour (X2)	P8 Tumour (X2)	P9 Tumour (X2)	P10 Tumour (X2)	P11 Tumour (X2)	P12 Tumour (X2)	P91 Tumour (X2)	P92 Tumour (X2)	P93 Tumour (X2)	P94 Tumour (X2)	P95 Tumour (X2)	P96 Tumour (X2)
**C**	P13 Tumour (X2)	P14 Tumour (X2)	P15 Tumour (X2)	P16 Tumour (X2)	P17 Tumour (X2)	P18 Tumour (X2)	P97 Tumour (X2)	P98 Tumour (X2)	P99 Tumour (X2)	P100 Tumour (X2)	P101 Tumour (X2)	P102 Tumour (X2)
**D**	P19 Tumour (X2)	P20 Tumour (X2)	P21 Tumour (X2)	P22 Tumour (X2)	P23 Tumour (X2)	P24 Tumour (X2)	P103 Tumour (X2)	P104 Tumour (X2)	P105 Tumour (X2)	P106 Tumour (X2)	P107 Tumour (X2)	P108 Tumour (X2)
**E**	P25 Tumour (X2)	P26 Tumour (X2)	P27 Tumour (X2)	P28 Tumour (X2)	P29 Tumour (X2)	P30 Tumour (X2)	P109 Tumour (X2)	P1110 Tumour (X2)	P111 Tumour (X2)	P112 Tumour (X2)	P113 Tumour (X2)	P114 Tumour (X2)
**F**	P31 Tumour (X2)	P32 Tumour (X2)	P33 Tumour (X2)	P34 Tumour (X2)	P35 Tumour (X2)	P36 Tumour (X2)	P115 Tumour (X2)	P116 Tumour (X2)	P117 Tumour (X2)	P118 Tumour (X2)	P119 Tumour (X2)	P120 Tumour (X2)
**G**	P37 Tumour (X2)	P38 Tumour (X2)	P39 Tumour (X2)	P40 Tumour (X2)	P41 Tumour (X2)	P42 Tumour (X2)	P121 Tumour (X2)	P122 Tumour (X2)	P123 Tumour (X2)	P124 Tumour (X2)	P125 Tumour (X2)	P126 Tumour (X2)
**H**	P43 Tumour (X2)	P44 Tumour (X2)	P45 Tumour (X2)	P46 Tumour (X2)	P47 Tumour (X2)	P48 Tumour (X2)	P127 Tumour (X2)	P128 Tumour (X2)	P129 Tumour (X2)	P130 Tumour (X2)	P131 Tumour (X2)	P132 Tumour (X2)
**I**	P49 Tumour (X2)	P50 Tumour (X2)	P51 Tumour (X2)	P52 Tumour (X2)	P53 Tumour (X2)	P54 Tumour (X2)	P133 Tumour (X2)	P134 Tumour (X2)	P135 Tumour (X2)	P136 Tumour (X2)	P137 Tumour (X2)	P138 Tumour (X2)
**J**	P55 Tumour (X2)	P56 Tumour (X2)	P57 Tumour (X2)	P58 Tumour (X2)	P59 Tumour (X2)	P60 Tumour (X2)	P139 Tumour (X2)	P140 Tumour (X2)	P141 Tumour (X2)	P142 Tumour (X2)	P143 Tumour (X2)	P144 Tumour (X2)
**K**	P61 Tumour (X2)	P62 Tumour (X2)	P63 Tumour (X2)	P64 Tumour (X2)	P65 Tumour (X2)	P66 Tumour (X2)	P145 Tumour (X2)	P146 Tumour (X2)	P147 Tumour (X2)	P148 Tumour (X2)	P149 Tumour (X2)	P150 Tumour (X2)
**L**	P67 Tumour (X2)	P68 Tumour (X2)	P69 Tumour (X2)	P70 Tumour (X2)	P71 Tumour (X2)	P72 Tumour (X2)	P151 Tumour (X2)	P152 Tumour (X2)	P153 Tumour (X2)	P154 Tumour (X2)	P155 Tumour (X2)	P156 Tumour (X2)
**M**	P73 Tumour (X2)	P74 Tumour (X2)	P75 Tumour (X2)	P76 Tumour (X2)	P77 Tumour (X2)	P78 Tumour (X2)						
**N**	P79 Tumour (X2)	P80 Tumour (X2)	P81 Tumour (X2)	P82 Tumour (X2)	P83 Tumour (X2)	P84 Tumour (X2)						

Figure S1. Example of layout.

Publisher's Note: MDPI stays neutral with regard to jurisdictional claims in published maps and institutional affiliations.

© 2021 by the authors. Licensee MDPI, Basel, Switzerland. This article is an open access article distributed under the terms and conditions of the Creative Commons Attribution (CC BY) license (http://creativecommons.org/licenses/by/4.0/).
